# Plakophilin-3 Binds the Membrane and Filamentous Actin without Bundling F-Actin

**DOI:** 10.3390/ijms24119458

**Published:** 2023-05-29

**Authors:** Jyoti Gupta, Erumbi S. Rangarajan, Regina B. Troyanovsky, Indrajyoti Indra, Sergey M. Troyanovsky, Tina Izard

**Affiliations:** 1 Cell Adhesion Laboratory, UF Scripps, Jupiter, FL 33458, USA; 2 Department of Dermatology, The Feinberg School of Medicine, Northwestern University, Chicago, IL 606112, USA; 3 The Skaggs Graduate School, The Scripps Research Institute, La Jolla, CA 92037, USA

**Keywords:** actin cytoskeleton, armadillo, desmosomes, phosphatidylinositol 4,5-bisphosphate, plasma membrane, plakophilin

## Abstract

Plakophilin-3 is a ubiquitously expressed protein found widely in epithelial cells and is a critical component of desmosomes. The plakophilin-3 carboxy-terminal domain harbors nine armadillo repeat motifs with largely unknown functions. Here, we report the 5 Å cryogenic electron microscopy (cryoEM) structure of the armadillo repeat motif domain of plakophilin-3, one of the smaller cryoEM structures reported to date. We find that this domain is a monomer or homodimer in solution. In addition, using an in vitro actin co-sedimentation assay, we show that the armadillo repeat domain of plakophilin-3 directly interacts with F-actin. This feature, through direct interactions with actin filaments, could be responsible for the observed association of extra-desmosomal plakophilin-3 with the actin cytoskeleton directly attached to the adherens junctions in A431 epithelial cells. Further, we demonstrate, through lipid binding analyses, that plakophilin-3 can effectively be recruited to the plasma membrane through phosphatidylinositol-4,5-bisphosphate-mediated interactions. Collectively, we report on novel properties of plakophilin-3, which may be conserved throughout the plakophilin protein family and may be behind the roles of these proteins in cell–cell adhesion.

## 1. Introduction

The armadillo repeat protein, plakophilin-3, is a multifunctional protein involved in cell–cell adhesion and signaling [1,2,3,4]. The most notable function of plakophilin-3, as well as two other plakophilins (plakophilin-1 and plakophilin-2) has been detected in desmosomes, the specialized intercellular adhesions found in various tissues. Their critical importance for these structures was illuminated through genetic experiments showing that the simultaneous knockout of plakophilin species in cells completely abolished desmosome formation [5,6,7]. Plakophilins also play a similarly crucial role in so-called composite junctions (for example, the junctions between cardiomyocytes), which combine the compositional and ultrastructural properties of desmosomes and adherens junctions [8,9,10]. Accordingly, mutations in plakophilin genes lead to skin and heart diseases, a common feature of impaired cell-cell adhesion [1,4,11].

The prevailing model Is that plakophilins serve as key linkers integrating major structural components of desmosomes. Such a scaffolding role of plakophilins is suggested by their direct interactions with all key desmosomal proteins. Specifically, plakophilins directly bind to (i) the cytosolic tails of the desmosomal receptors desmogleins and desmocollins; (ii) the desmosomal plaque proteins plakoglobin and desmoplakin; (iii) the intermediate filament proteins known as keratins; and (iv) the adherens junction protein β-catenin [4,12,13,14]. Through alternative models, plakophilins control the delivery of desmosomal proteins to assembly sites or some nonidentified but critical signaling events [3,15].

Beyond their desmosome localization, we recently showed that plakophilin-3 resides in the most apical segment of the lateral cell plasma membrane [7]. This extra-desmosomal location of plakophilin-3 in the apicolateral cell cortex depends on the activity of the defective partitioning protein 3 polarity complex. It may be important for the polarized distribution of desmosomes in simple epithelia, thereby implicating plakophilin-3 in apicobasal cell polarity mechanisms. In addition to their role in adhesion, plakophilins participate in many other processes ranging from transcription regulation and mRNA stability to epidermal-growth-factor-receptor-, Rho-, and protein-kinase-C-dependent signaling [1,3,4]. The data show that plakophilins, similar to many armadillo-repeat-motif-containing proteins, couple cell-cell adhesion with various cellular activities. Despite such interesting and important functions, the exact role of plakophilins in desmosomes and extra-desmosomal cortical, cytosolic, and nuclear pools remains to be studied.

One of the prominent strategies used to identify the molecular mechanisms of the functions of plakophilins is to gain a detailed understanding of their structure. Plakophilins are members of a p120-catenin subfamily of armadillo repeat motif proteins [16,17,18]. This distinctive feature, as determined by crystallography of p120-catenin and plakophilin-1 [19,20], is the nine armadillo repeat motif organization of the armadillo domain interrupted between repeats four and five by an insert of approximately 60 amino acids. The p120-catenin subfamily is subdivided into two groups. The proteins of the p120-catenin group include p120-catenin itself and catenins-δ1 and -δ2, plakophilin-4, and ARVCF (the armadillo repeat gene deleted in Velo-Cardio-Facial syndrome) and are localized to adherens junctions. The armadillo repeat motif domains of these proteins interact with a juxta membrane domain of classic cadherins. Plakophilin isoforms 1, 2, and 3 are more distant from p120-catenin and do not show interactions with classic cadherins [3]. Apart from the armadillo domains of other catenins, including those of p120-catenin and β-catenin, which serve as key hubs mediating critical protein-protein interactions [18], the binding that is specific to the armadillo domain of plakophilins in desmosomes or the cell cortex has not been identified. The sites interacting with all known desmosome- or cell-cortex-associated plakophilin partners, such as desmogleins, desmocollins, plakoglobin, desmoplakin, keratins, β-catenin, and stratifin, have been mapped to the amino-terminal unstructured domain of plakophilins [4,21,22]. Surprisingly, this unstructured region, apart from a short region close to the amino terminus, exhibits no sequence similarities between plakophilin species [3]. While the key partners of the armadillo repeat motif domain of plakophilins involved in desmosome assembly remain to be identified, this domain is important for plakophilin function in desmosomes, since the head domain of any plakophilin cannot maintain desmosome formation alone [21,23].

To better understand the function of plakophilins, we investigated the structure and function of plakophilin-3 and discovered new interactions with the actin cytoskeleton and phospholipids. These two interactions may potentially target plakophilin-3 in the cell cortex. While plakophilin-3 shares the nine armadillo repeat motifs described above for the plakophilin-1 and the truncated plakophilin-2 crystal structures, our cryogenic electron microscopy (cryoEM) structure of plakophilin-3 reveals an important new structural insights. Unlike the plakophilin-1 and plakophilin-2 structures, the carboxy terminus of plakophilin-3 is fully resolved in our cryoEM structure, and the carboxy-terminal α-helix (residues 790-796) appears to form a cap on the last armadillo repeat motif. Notably, we found that plakophilin-3 directly interacts with actin filaments and phospholipids. The recruitment of plakophilin-3 to actin bundles, specifically to the sites of their interactions with adherens junctions, was confirmed via fluorescence microscopy. These two new binding activities of the armadillo repeat motif domains of plakophilin-3 may have crucial functions in desmosome assembly and apicobasal cell polarity.

## 2. Results

### 2.1. Plakophilin-3 cryoEM Structure

**Figure 1:**
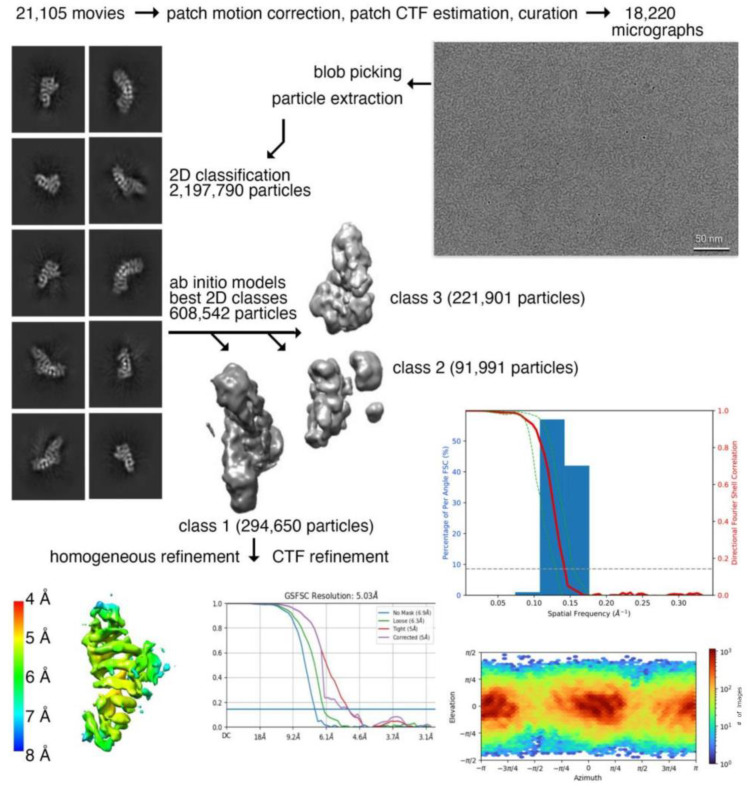
**CryoEM data processing workflow for plakophilin-3.** Motion-corrected cryoEM micrograph for plakohilin-3 (**top**, **right**). Representative 2D class averages of monomeric plakophilin-3 used for 3D reconstruction (**top**, **left**). High-quality micrographs (18,220) were selected after motion correction and used for the selection of 2,197,790 particles. Multiple rounds of 2D classification were performed to remove noise, yielding 608,542 plakophilin-3 particles, which were subjected to ab initio classification. One out of three classes, class 1, containing 294,650 particles, revealed structural features for all nine armadillo repeat motifs of plakophilin-3 that were subjected to 3D refinement and contrast transfer function (CTF) refinement. (**Middle**, **right**) Directional Fourier shell correlation plot for plakophilin-3. The red line shows the global Fourier shell correlation (FSC), the green lines show the directional resolution spread values defined according to ±1 standard deviation from the mean of the directional resolutions, and the blue bars represent a histogram of 100 directional resolutions evenly sampled over the 3D FSC. A sphericity of 0.951 was determined at an FSC threshold of 0.5, which indicates significant isotropic angular distribution. (**Bottom left**) The resolution of the map was determined based on local resolution estimation in cryoSPARC. The orientation distribution plot obtained from cryoSPARC is shown at the **bottom** on the **right**.

**Figure 2:**
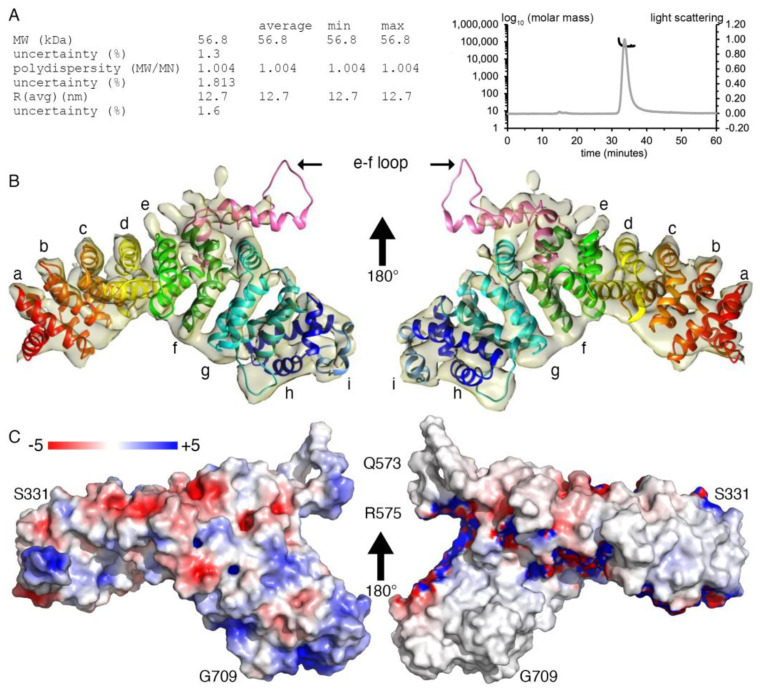
**CryoEM structure of the armadillo repeat motif domain of plakophilin-3.** (**A**) The multi-angle light scattering size exclusion statistics obtained for plakophilin-3 are provided in the table (**left**). The profiles of the light scattering (gray) and the calculated molar mass (black) are as shown (**right**). (**B**) Back and front views of a cartoon drawing of our cryoEM plakophilin-3 structure (residues 305-797) and final Coulomb potential map. Each armadillo repeat motif is labeled (a–i), and the loop between the fifth (e) and sixth (f) armadillo repeat motifs is indicated. (**C**) Back and front views of the electrostatic surface potential representation of our plakophilin-3 cryoEM structure. The electrostatic potential gradient is from −5 to +5 *k*B*T* (red, negative; blue, positive), where *k*B is the Boltzmann constant, and *T* is the temperature. Some residues are labeled.

Our 57 kDa monomeric plakophilin-3 (residues 305-797) cryoEM structure (Figure 1 and Figure 2) is one of the smaller cryoEM structures determined to date [24]. Accomplishing a meaningful resolution of such a small protein required a large dataset, which allowed for the strict curation of the initial raw movies and subsequent curation through several rounds of 2D classification to select suitable particles (Figure 1). The overall completeness of the particle orientations was analyzed by 3D Fourier shell correlation analyses [25,26], which showed a sphericity value of 0.951 (Figure 1), suggesting a near-complete isotropic behavior of the particles, which further confirms the absence of any preferred orientation. The fit for all nine armadillo repeat motifs into the Coulomb potential map was optimal when flipped (Table 1). The residues in the loop region did not show continuous density and could not be built with certainty. However, we observed an additional density in the loop region between the fifth and sixth armadillo repeats, e and f. The limitations of the resolution and missing density might be due to the flexibility of the loop, which is primarily disordered except for two short α-helices. Noticeably, during data processing, we also observed that 36% of the particles were segregated into a separate class, which resembled half of a plakophilin-3 molecule. We have removed these particles to obtain an interpretable isotropic map that showed a reasonable orientation distribution and a sphericity value of 0.951, which suggests that the particles do not exhibit a preferred orientation.

**Figure 3:**
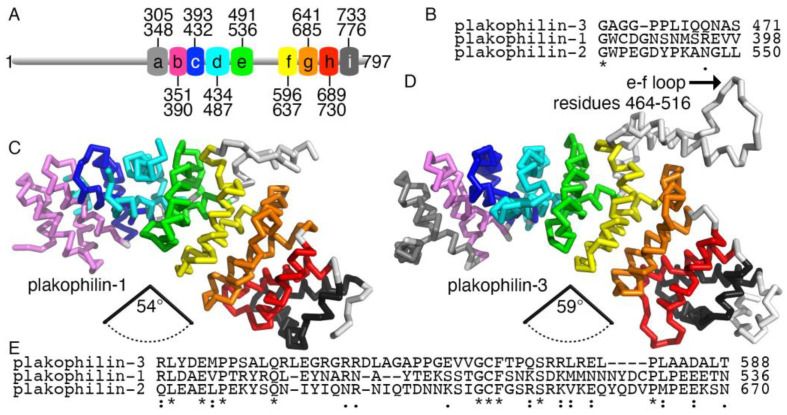
**Plakophilin-3 is less compact than plakophilin-1.** (**A**) Schematic domain structure of plakophilin-3 showing the nine armadillo repeat motifs and residue boundaries. (**B**) The insert in the fourth armadillo repeat motif is disordered in the plakophilin-1 structure and has a distinct sequence. (**C**) The human plakophilin-1 crystal structure (PDB entry 1xm9) [19] is more compact compared to (**D**) our plakophilin-3 cryoEM structure. In the plakophilin-1 structure, the angle between the Cα of residues 277, 428, and 586 is 54°. The angle between the equivalent Cα of residues 351, 501, and 668 in plakophilin-3 is 58.6°. (**E**) Amino acid sequence alignment of the three human plakophilin insert regions shows that plakophilin-3 is distinct in that region. The asterisk, colon, and period symbols below the sequence each represent identity, and high and low conservation, respectively.

As seen for the armadillo repeat motif domain of plakophilin-1 (PDB entry 1xm9) [19] and plakophilin-2 (PDB entry 3tt9) [27], our plakophilin-3 structure has nine armadillo repeat motifs with an additional carboxy-terminal α-helix that forms a half-donut-shaped domain (Figure 2 and Figure 3). An insert is found between the fifth and sixth armadillo repeat motifs, the “e-f loop” (Figure 2B), that projects outwards without interrupting the interactions of the neighboring armadillo repeat motifs (Figure 2 and Figure 3D). As seen in other armadillo repeat motif structures, three α-helices fold back to form the nearby amino and carboxy termini.

Plakophilin-3 is highly conserved among species [28]. Human isoforms 1 and 2 share ~34% of their sequences, while these plakophilin isoforms are less similar to plakophilin-3 (~29% identity). This trend is reversed in terms of sequence similarity. Plakophilin isoforms 1 and 2 are less similar to each other (approximately 44% sequence similarity) and more similar when comparing each to plakophilin-3 (approximately 54%) [29]. This is also the case when comparing the armadillo repeat domains (~61% and ~52%, respectively). However, the insert of plakophilin-3 (residues 538-596) has less identity with the other isoforms (24-27%) compared to the 37% sequence identity between the inserts of plakophilin-1 and plakophilin-2 (Figure 3B). The insert is disordered in the plakophilin crystal structures. Notably, the isoelectric point of the inserts differs, with plakophilin-3 being the most basic (pI of 8.2) and plakophilin-1 the most acidic (pI of 4.9) (Figure 3E). Plakophilin-2 is closer to neutral (pI of 6.4).

Our plakophilin-3 cryoEM structure (Figure 2 and Figure 3) is similar to the plakophilin-1 (PDB entry 1xm9) [19] and truncated plakophilin-2 (PDB entry 3tt9) [27] crystal structures. These crystal structures can be superimposed onto our plakophilin-3 structure with root mean square deviations of 1.68 Å for 2,289 plakophilin-1 atoms and 0.875 Å for 1,078 plakophilin-2 atoms. Therefore, we will not repeat the detailed structural analyses and description published for the other plakophilin isoforms and, instead, will focus on discussing the novelty of our plakophilin-3 structure.

The fourth armadillo repeat motif of plakophilin-3 is unique. A larger loop connects the first and last α-helices, which are disordered in plakophilin-1 (residues 385-399). The middle α-helix seen in the plakophilin-2 crystal structure (residues 493-507) seems to be missing in plakophilin-3 (residues 458-468). This structural difference is also reflected in the lack of amino acid similarity (Figure 3B). Despite plakophilin-3 exhibiting an overall resemblance with the armadillo repeat domains of plakophilin-1, plakophilin-2, and p120-catenin, the curvature of the domain seems to be dictated by the arrangement of the third α-helix from the nine armadillo repeats, which is slightly open compared to the other structures from its subfamily (Figure 3C,D). This is relevant since it represents the putative interacting surface with different binding partners. This may be attributed to the relative positioning of the last α-helix in the insert preceding the armadillo repeat motif f (Figure 3C,D).

Our plakophilin-3 structure comprises the longest polypeptide chain of this family. It harbors one additional α-helix at the amino terminus and two additional carboxy-terminal α-helices compared to the plakophilin-1 crystal structure. The truncated plakophilin-2 structure also misses the amino-terminal α-helix seen in our structure and is truncated before the insert, after the fifth armadillo repeat motif, thus missing the last four armadillo repeats.

### 2.2. Plakophilin-3 Forms a Dimer and Monomer

**Figure 4:**
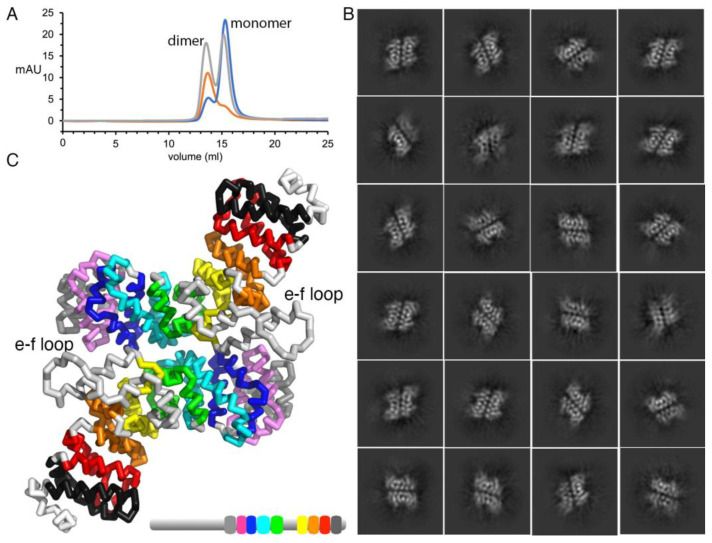
**Plakophilin-3 is a monomer and a dimer.** (**A**) Size exclusion chromatogram for plakophilin-3. The apparent molecular weights for the plakophilin-3 monomer and dimer were found to be 60.25 kDa and 120.6 kDa, respectively, compared to the calculated molecular weights of 57.2 and 114.4 kDa. Initial plakophilin-3 chromatogram in gray is normalized. Fractions from the first peak that correspond to the dimer were pooled and reloaded, red. Fractions from the second peak that correspond to the monomer were pooled and reloaded, blue. (**B**) Representative 2D class averages of the particles for the plakophilin-3 dimer are shown. (**C**) The plakophilin-3 dimer model based on the 2D class averages suggests that the loop between armadillo repeat motifs e and f is near the dimer interface.

While we could not generate full-length plakophilin-3 due to protein precipitation, we successfully produced the carboxy-terminal armadillo domain without the unstructured 304 amino-terminal residues. When we purified plakophilin-3, we obtained a mixture of monomers and dimers, as analyzed by size exclusion chromatography (Figure 4A). At higher concentrations and lower reducing conditions, the equilibrium shifted towards the dimer. However, in the presence of reducing agents such as dithiothreitol or tris(2-carboxyethyl)phosphine, we obtained mainly monomeric plakophilin-3. This was further confirmed using multi-angle light scattering data (Figure 2A). We obtained a single peak corresponding to a molar mass of 56.8 (±1.3%) kDa and a radius of gyration of 12.7 (±1.6%) nm. Interestingly, while the monomeric form seems to be predominant, we obtained 2D class averages corresponding to a dimer from 43% of all the particles that were not triaged (Figure 4B). Other armadillo-repeat-containing proteins exhibit concentration-dependent dimerization and even higher-order oligomerization [30].

The 2D class averages indicated the formation of plakophilin-3 homodimers. However, the 2D classes obtained were highly monotonous, providing only the side-by-side arrangement of two protomers, indicating a robust preferred orientation for the dimer particles. Further attempts at 3D reconstruction using *ab initio* modeling and homogeneous refinement yielded no interpretable volume. Hence, the 2D class average was used as a template to manually superpose two plakophilin-3 molecules to obtain the plakophilin-3 homodimer model. Interestingly, the large inset between armadillo repeats e and f is at the interface of the two subunits, suggesting a possible role in mediating the dimerization. This arrangement might allow for interaction with two binding partners simultaneously to form a multi-layer complex at or near the desmosomes.

### 2.3. Plakophilin-3 Binds to the Membrane

**Figure 5:**
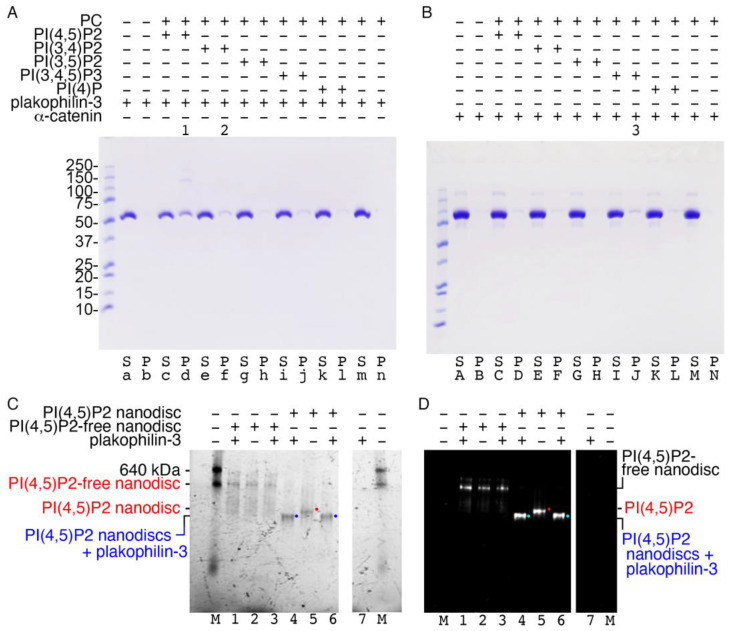
**Plakophilin-3 binds to the membrane.** (**A**) Plakophilin-3 binds to PI(4,5)P_2_-containing vesicles (**lane d**, indicated by “1”), which results in some dimerization, as determined by co-sedimentation. Plakophilin-3 remains in solution in the absence of lipids (**lane a**). **Lane f** (indicated by “2”) shows weak plakophilin-3 binding to PI(4,5)P_2_. (**B**) Our α-catenin control (residues 82-906) weakly binds PI(3,4,5)P_2_-containing vesicles (**lane J**, indicated by “3”), as reported [31]. S, supernatant; P, pellet; PC, phosphatidylcholine; PI(4,5)P2, phosphatidylinositol 4,5-bisphosphate; PI(3,4)P2, phosphatidylinositol 3,4-bisphosphate; PI(3,5)P2, phosphatidylinositol 3,5-bisphosphate; PI(3,4,5)P3, phosphatidylinositol 3,4,5-trisphosphate; PI(4)P, phosphatidylinositol 4-phosphate. Similar results were obtained from two independent experiments. (**C**,**D**) Native gel shift assay to detect the binding of plakophlin-3 to fluorescently labeled PI(4,5)P_2_-free and PI(4,5)P_2_-containing nanodiscs, as detected by (**C**) ultraviolet activation and (**D**) rhodamine fluorescence. **Lanes 1–3**, Plakophilin-3 showed no binding to the PI(4,5)P_2_-free nanodisc. **Lanes 4–6**, Binding of plakophilin-3 was observed for the PI(4,5)P_2_ nanodisc (blue or cyan dots, complex; red dots, unbound nanodiscs). **Lanes 7**, plakophilin-3 does not run into the native gel due to its basic pI; M, molecular weight markers. Data are representative of two independent experiments.

Deletion mutagenesis studies of plakophilin-1 determined that its carboxy terminus, residues 686-726, are necessary to recruit plakophilin-1 to the plasma membrane [21]. Therefore, we questioned whether plakophilin-3 is also a membrane-associated protein. To test our hypothesis, we performed a lipid vesicle co-sedimentation assay (Figure 5A and 5B). We used α-catenin, which has been shown to weakly bind PI(3,4,5)P_3_ [31], as a control (Figure 5B). Given that the readout of the standard vesicle pull-down assay is the pelleting of the membrane-binding protein with the lipid vesicle, we first confirmed that our proteins did not precipitate in the absence of lipids. Plakophilin-3 sedimented in the presence of phosphatidylinositol 4,5-bisphosphate (PI(4,5)P_2_) as a monomer or dimer (Figure 5A) whereas α-catenin weakly bound PI(3,4,5)P_3_ (Figure 5B). Our native gel shift assays with nanodiscs corroborated the binding of plakophilin-3 to PI(4,5)P_2_-containing nanodiscs (Figure 5C,D). Notably, the overall electrostatic surface potential of plakophilin-3 projects a cluster of residues forming a basic patch at the carboxy terminus and an additional acidic patch on the large insert between armadillo repeats e and f. The orientation of these basic patches suggests possible coordinated involvement in membrane attachment.

### 2.4. Plakophilin-3 Binds Actin Filaments but Does Not Bundle F-Actin

**Figure 6:**
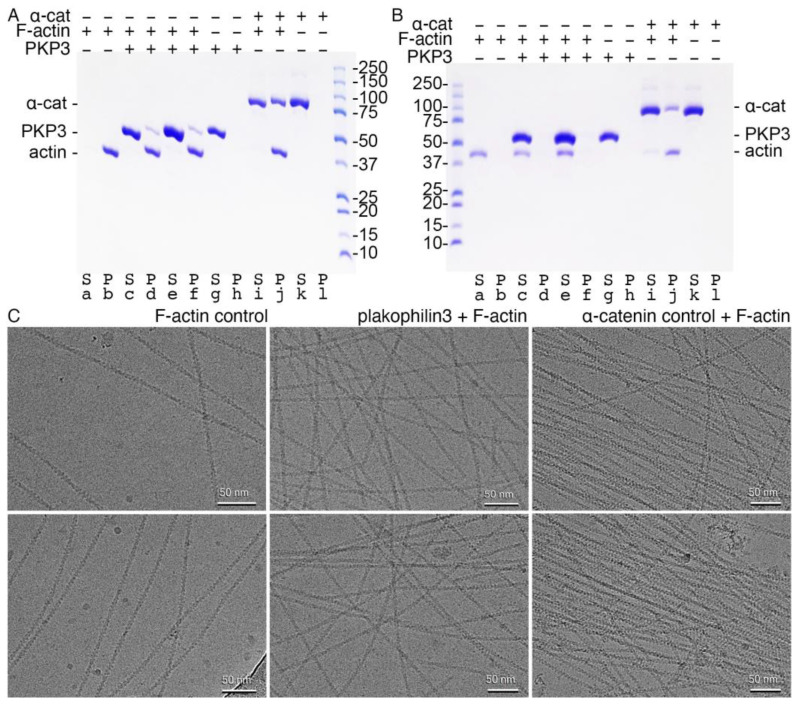
**Plakophilin-3 binds filamentous actin without bundling F-actin**. (**A**) High-speed centrifugation to detect F-actin binding. Plakophilin-3 co-sediments with F-actin (**lanes d, f**). Plakophilin-3 remains in solution in the absence of F-actin (**lane g**). Actin is fully polymerized (**lane b**). Our α-catenin control binds F-actin (**lane j**) and remains in solution in the absence of actin (**lane k**). Similar results were obtained from two independent experiments. (**B**) Low-speed centrifugation to detect F-actin bundling. Plakophilin-3 does not bundle F-actin (**lane e**). Purified actin (**lane a**), plakophilin-3 (**lane g**), and α-catenin (**lane k**) are soluble. Our α-catenin control bundles F-actin (**lane j**). (**C**) CryoEM micrographs. **Left**, F-actin alone. **Middle**, F-actin in the presence of plakophilin-3. **Right**, F-actin in the presence of α-catenin. Top and bottom panels are independent repeats.

The overexpression of the armadillo repeat motif domain of plakophilin-1 has been shown to colocalize with actin-enriched structures such as filopodia and other cell protrusions [13]. Therefore, it seems plausible that plakophilin-3 is likewise a cytoskeletal protein. We thus tested whether the armadillo repeat domains of plakophilin-3 directly interact with F-actin by performing actin-bundling and -binding co-sedimentation assays. We used α-catenin as a positive control. Given that the readout of the standard actin co-sedimentation assay is the pelleting of the cytoskeletal protein with filamentous actin, we confirmed that our proteins did not precipitate in the absence of F-actin (Figure 6A,B). Our actin-binding studies also demonstrated that actin was fully polymerized. Once we established that our proteins remained soluble upon high-speed centrifugation, which allowed for an unambiguous interpretation of our results, we performed our actin co-sedimentation assay at a high centrifugal speed. We found that plakophilin-3 and α-catenin bind F-actin (Figure 6A).

Next, we determined whether plakophilin-3 bundles F-actin by performing our actin co-sedimentation assay at a low centrifugal speed (Figure 6B). As with our F-actin-binding experiment, we first confirmed that our proteins do not precipitate without F-actin. Once we established that our proteins remained soluble upon centrifugation, we performed our actin bundling assay at a lower speed than the previous centrifugal speed. At a 1:2 F-actin to plakophilin-3 molar ratio, we found that plakophilin-3 does not bundle F-actin. In contrast, our control protein, α-catenin, readily bundled F-actin. These results were corroborated by cryoEM (Figure 6C).

### 2.5. Plakophilin-3 Forms Actin-Associated Clusters in the Vicinity of Adherens Junctions

**Figure 7:**
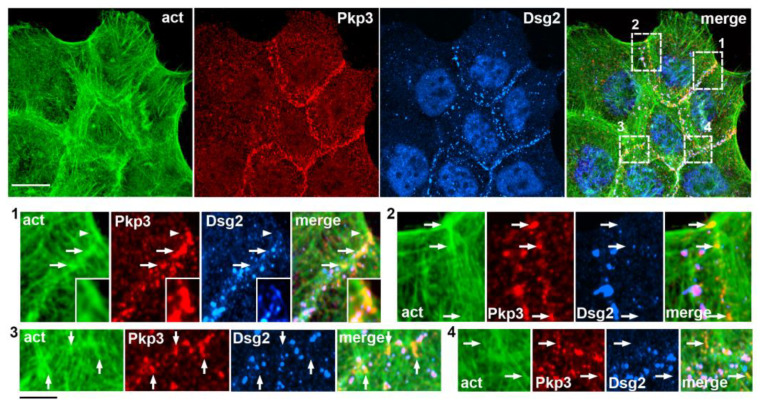
**Extra-desmosomal plakophilin-3 clusters are recruited to cell–cell junction-associated actin bundles.** Triple-fluorescence microscopy of A431 cells stained for actin filaments (act, green), plakophilin-3 (Pkp3, red), and the desmosomal cadherin, desmoglein-2 (Dsg2, blue). The upper micrographs show a low magnification of a group of cells. Scale bar, 25 μm. Zoomed images of four representative cell-cell contact regions that are designated with dashed boxes (numbered 1 to 4) are in the bottom rows. Note that desmoglein-2 and plakophilin-3 are colocalized in desmosomes. In addition to desmosomes, each of the selected regions contains several plakophilin-3-positive clusters of different sizes (some of them are indicated with arrows), which are *desmoglein-2*-deficient but align along the actin-enriched structures. One of them (marked by the arrowhead in the region #1) is shown zoomed-in in the insets. Note that this large plakophilin-3 cluster incorporates several small desmosomes. Bars, 7 μm.

To test whether the endogenous plakophilin-3 interacts with the actin cytoskeleton in cells, we co-stained epithelial A431 cells for plakophilin-3, F-actin, and a key desmosomal cadherin, desmoglein-2. As expected, an inspection of the obtained images showed that the desmoglein-2-marked desmosomes were also positive for plakophilin-3 staining (Figure 7). As we also expected, the desmosomes showed no specific actin enrichment. At the same time, some F-actin-containing structures could always be found in the desmosome vicinity, suggesting that some interactions between desmosomes and actin filaments might occur. More importantly, the cells also exhibited many cell-cell-contact-associated plakophilin-3 clusters devoid of desmoglein-2 (arrows in zoomed areas 1–4 in Figure 7) and, therefore, could not represent desmosomes. These variations in the size of the *desmoglein-2*-deficient plakophilin-3 clusters were nearly always detected at the ends of the junction-associated radial actin bundles. Such a location is typical for adherens junctions in A431 cells [32]. Interestingly, some of these plakophilin-3 clusters incorporated one or several barely visible desmosomes, as shown in the magnified plakophilin-3 cluster in the inset of the zoomed area #1 in Figure 7. We found no plakophilin-3-specific staining in any other actin-enriched structures in the A431 cells, including cell protrusions and focal adhesions.

**Figure 8:**
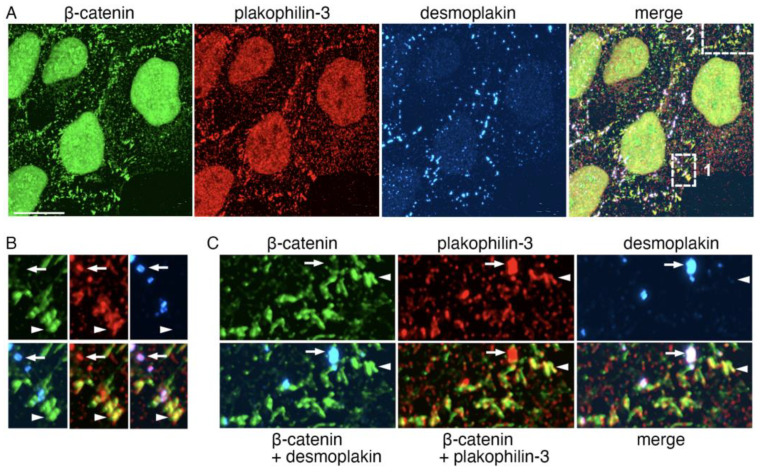
**Extra-desmosomal plakophilin-3 clusters are associated with adherens junctions**. (**A**) Triple-fluorescence microscopy of images A431 cells stained for plakophilin-3 (red) in combination with the constitutive proteins of adherens junction and desmosomes, β-catenin (green) and desmoplakin (blue), respectively. The upper micrographs show cells at a low magnification. Bar, 25 μm. Two representative cell–cell contact regions that are demarcated with dashed boxes (1 and 2) are shown zoomed-in in the bottom rows (bar, 7 μm) as single-stained images (β-catenin, plakophilin-3, and desmoplakin) in (**B**) or in three different combinations (β-catenin + desmoplakin; β-catenin + plakophilin-3; and merge) in (**C**). Note that both the desmoplakin-labeled desmosomes (some of them marked by arrows) and β-catenin-labeled adherens junctions (arrowheads) contain plakophilin-3. Additionally, note that the localizations of β-catenin and plakophilin-3 in the adherens junctions do not exactly match one another.

To verify that the *desmoglein-2*-deficient clusters were colocalized to adherens junctions, we stained A431 cells for plakophilin-3 together with the markers of adherens junctions and desmosomes, β-catenin and desmoplakin, respectively (Figure 8). This triple staining confirmed the dual localization of plakophilin-3. Most desmosomes, marked by desmoplakin (arrows in Figure 8), contained plakophilin-3 but not β-catenin. Similarly, nearly all the adherens junctions (arrowheads) exhibited variations the in size and brightness of the plakophilin-3 clusters (but not desmoplakin), which only partially matched one another. Overall, our immunomorphological analyses showed that adherens junctions, known to be abundant in short-lived actin filaments [32], are significant sites for the different desmosomal pools of plakophilin-3.

## 3. Discussion

Plakophilins compose a group of armadillo repeat motif domain proteins that play different roles in cells, including an essential one in forming desmosomes. While it remains unclear how plakophilins facilitate desmosome assembly, defects in this function cause severe cardiological and skin diseases. The fact that plakophilin deficiency completely abolishes desmosomes [5,6,7] suggests that these proteins are required for the initial events in desmosome assembly. The available data also show that desmosome assembly starts with desmosomal cadherins and desmoplakin clustering. The formation of these initial clusters relies on the actin cytoskeleton but not on intermediate filaments, which anchor matured desmosomes [33,34,35,36,37]. Finally, plakophilins, even though they interact with all known desmosomal proteins and intermediate filaments [3,38], can be recruited to the cell cortex without these interactions [7,12,37]. In line with these reports, through triple-fluorescence microscopy studies using A431 cells, we show that plakophilin-3 is located near the actin-enriched cell-cell junctions known as adherens junctions. Future studies might identify the interactions which target plakophilin-3 in adherens junctions and whether this targeting is essential for the assembly of desmosomes. Here, we report several novel features of plakophilin-3 which might facilitate the recruitment of this protein into the cell cortex and, therefore, could play key roles in early events of the assembly of desmosomes.

We present the cryoEM structure plakophilin-3, consisting of the entire armadillo repeat motifs domain, although the loop region lacks continuous density in the Coulomb map. As a member of the p120-related armadillo repeat subfamily proteins, the overall arrangement of the armadillo repeat motifs domain of plakophilin-3 resembles the crystal structures of plakophilin-1 [19] (PDB entry 1xm9) and p120-catenin [20] (PDB entry 3l6x). Plakophilin-3 and plakophilin-1 do not have an additional loop between the fourth and fifth armadillo repeats e and f, as compared to p120-catenin, which retains this loop. Interestingly, our plakophilin-3 cryoEM structure shows a bent curvature with the deviation occurring immediately after the e-f loop region between the fifth and sixth repeats g and h, similar to plakophilin-1 and p120-catenin. However, in comparison, plakophilin-3 is slightly less curved. In contrast, the β-catenin armadillo repeat motif structure has a relatively straight orientation compared to the plakophilin-1 crystal structure due to the large insert between repeats 5 and 6 [19]. Such a conformation can also be seen in our plakophilin-3 cryoEM structure.

Furthermore, plakophilin-3 harbors a fully resolved smaller loop between the second and third α-helices of the fifth repeat “e”, which is much longer (by approximately ten amino acids) in plakophilin-1 and p120-catenin and was not observed in these crystal structures. The ability to form homodimers provides an additional dimension to the plakophilin-3 interactions at cell junctions. This will be an exciting area of research for future studies.

The 2D class averages derived from the plakophilin-3 dimer gel filtration peak indicated the presence of homodimers in solution. However, the homodimers showed a robust preferred orientation, which significantly hampered our efforts to produce a 3D reconstruction. Nevertheless, clear features depicting the overall arrangement of α-helices and an apparent two-fold symmetry in the 2D class averages provided enough template to manually assemble the homodimer model using our plakophilin-3 monomer 3D-reconstructed structure. The arrangement suggests the proximity of the large insert region at the dimeric interface. The proposed involvement of the insert region in the homodimer formation is evident. The ability of plakophilin-3 to transition from a monomer to a dimer could play a role in regulating the stability of the desmosomal complex within the cellular context. Additionally, excess plakophilin-3 might be stored in the form of homodimers to participate either in aiding nuclear signaling or in extra-desmosomal assemblies, including adherens junctions. Such dimerization of plakophilin-3 is similar to the human αE-catenin in adherens junctions, which functions as a monomer in the E-cadherin–β-catenin–αE-catenin complex and as homodimers in the cytosol [39,40].

An important new feature of the plakophilin-3 armadillo repeat motif domain is its direct binding to actin filaments. We showed that some pools of plakophilin-3 are associated with the termini of actin bundles attached to adherens junctions in A431 cells. This pool of plakophilin-3 is distinct from the desmosome-associated pool of the same protein in these cells. It could be related to the recently identified extra-desmosomal pool of plakophilin-3, located in the multicellular junctions of polarized DLD1 cells [7]. This finding is consistent with the previous observation that the overexpressed armadillo repeat motifs domain of plakophilin-1 is associated with actin-enriched lamellipodia and stress fibers [13]. However, the report showed no direct interactions between the plakophilin-1 armadillo repeat motifs domain and monomeric actin. To further delineate the nature of the plakophilin-3 interaction with the actin cytoskeleton, we conducted high-speed and low-speed co-sedimentation analyses with purified proteins. Under our experimental conditions, the armadillo repeat motifs domain of plakophilin-3 exhibited definitive binding to F-actin, albeit at a lower affinity. It failed to bundle the actin filaments in low-speed conditions, corroborating our colocalization results obtained with A431 cells. Additional studies are needed to understand whether the direct interaction of plakophilin-3 with F-actin is involved in the mechanisms of desmosome assembly and the reported anchorage of different desmosomal proteins to the actin cytoskeleton [35,36].

The second important feature of the plakophilin-3 armadillo repeat motifs domain that we determined is its interaction with phosphatidylinositol phosphates. Lipid co-sedimentation analyses indicated a clear preference of PI(4,5)P_2_ for plakophilin-3. The interaction with PI(4,5)P_2_ is a well-characterized mechanism of recruitment of different proteins to the plasma membrane [41]. Furthermore, the actin-binding activities of several actin-binding proteins (e.g., talin, vinculin, proteins from ezrin/radixin/moesin family) were shown to be regulated by binding to PI(4,5)P_2_-conatining membrane clusters [42,43,44,45,46,47,48]. Our observation suggests that plakophilin-3 might recruit other proteins to the plasma membrane at either desmosomes or adherens junctions. Earlier studies showed that plakophilin-1 engages with the plasma membrane through its armadillo repeat motifs domain, especially its carboxy-terminal 40 amino acids encompassing residues 686-726 [21].

Similarly, due to sequence conservation, it is expected that plakophilin-2 and plakophilin-3 might bind the plasma membrane through their carboxy terminus within the armadillo repeat motifs region. PI(4,5)P_2_ binding might involve a cluster of basic residues available in both the carboxy terminus and the large insert region (between the fifth and sixth armadillo repeats) within the armadillo repeat motifs domain of plakophilin-3. It may act either in tandem or individually to aid recruitment to the plasma membrane. Interestingly, the only specific function attributed to the insert is its palmitoylation, which was shown to be essential for plakophilin targeting of the plasma membrane [49].

Collectively, the multitude of interactions exhibited by plakophilin-3 in binding to PI(4,5)P_2_, F-actin, and itself through dimerization provides attractive functions within the cellular context at both desmosomes and adherens junctions. These interactions stabilize cell-cell contact and help to recruit other structural desmosomal or adherens junction proteins to the sites of cell-cell interactions. Such knowledge about the exact boundaries involved in these interactions and their mechanisms will significantly aid in the advancement of our understanding of the functions of cell junctions.

## 4. Materials and Methods

### 4.1. Cloning

Full-length human plakophilin-3 (accession No. Q9Y446, obtained from Dr. Katheen Green [14]) was cloned in pET28 with eight histidine residues followed by the HRV-3C site at the amino terminus. Residues 305-797 were then subcloned into pET28 with eight histidine residues, followed by the HRV-3C site at the amino terminus. All clones were prepared by Dr. Oskar Laur (Emory University).

### 4.2. Protein Purification

Plakophilin-3 (residues 305-797) was expressed in the *Escherichia coli* strain BL21(DE3) Rosetta2 (Novagen, Reno, NV, USA) at 25 °C for 20 h. Cells were induced for protein expression using 1 mM isopropyl β-D-1-thiogalactopyranoside. The cells were harvested by centrifugation at 5000× *g* for 25 min and lysed by sonication in lysis buffer (20 mM Tris, pH 8, 500 mM NaCl, containing two ethylenediaminetetraacetic acid-free protease inhibitor cocktail tablets). The lysate was clarified at 35,000 rpm for 30 min. The supernatant was loaded onto a Ni-nitriloacetic acid column equilibrated with lysis buffer, and the protein was eluted with a linear gradient of imidazole using the AKTA fast protein liquid chromatography system. The fractions containing plakophilin-3 were concentrated with 5% glycerol, and the aliquots were frozen in liquid nitrogen and stored at −80 °C. Before usage, the thawed protein fractions were subjected to a Superdex S-200 10/300 size exclusion column equilibrated with 20 mM Tris (pH 7.5), 150 mM NaCl, and 1 mM dithiothreitol. The production and purification of αE-catenin (residues 82-906) were carried out as described earlier [50].

### 4.3. Size Exclusion Chromatography and Multi-Angle Light Scattering

Size exclusion chromatography and multi-angle light scattering (SEC-MALS) analyses were carried out with purified plakophilin-3, as described previously [51,52]. Briefly, protein samples were passed through a Superdex 200 10/300 GL column, pre-equilibrated with 20 mM Tris (pH 7.5), 150 mM NaCl, and 1 mM dithiothreitol, using an Agilent 1260 Infinity series HPLC system connected with a Dawn-Heleos II multi-angle light-scattering detector (Wyatt Technology) and OptiLab T-rex differential refractive index detector (Wyatt Technology). Bovine serum albumin at 2 mg/mL was used as a standard, and all data acquisition and analyses were carried out using ASTRA software version 6.1.

### 4.4. Lipid Vesicle Co-Sedimentation

Lipid binding to plakophilin-3 was assayed as described previously [46]. Briefly, lipid vesicles of phosphatidylcholine (PC), phosphatidylinositol 4,5-bisphosphate (PI(4,5)P2), phosphatidylinositol 3,4-bisphosphate (PI(3,4)P2), phosphatidylinositol 3,5-bisphosphate (PI(3,5)P2), phosphatidylinositol 3,4,5-trisphosphate (PI(3,4,5)P3), and phosphatidylinositol 4-phosphate (PI(4)P) were prepared in chloroform (or chloroform/methanol/water at a molar ratio of 1:2:0.8 for phosphatidylinositol phosphates, as recommended by Avanti Polar Lipids) to a final composition of 95% chicken egg PC and 5% PI(4,5)P_2_, PI(3,4)P_2_, PI(3,5)P_2_, PI(3,4,5)P_3_, and PI(4)P (Avanti Polar Lipids). The lipid mixtures were dried under an argon stream and resuspended in resuspension buffer (20 mM Tris (pH 7.5), 400 mM NaCl, 0.1 mM ethylenediaminetetraacetic acid, and 2 mM dithiothreitol). Small unilamellar vesicles were then produced by sonication after incubating at 37 °C for 60 min. Samples containing 50 μg of total lipid in 20 μL suspension and 3.5 μM plakophilin-3 protein were incubated at 4 °C for 1 h, followed by centrifugation at 32,500 rpm for 15 min. The supernatant and pellet were separated carefully, and the pellet was washed twice and resuspended in 20 μL resuspension buffer.

### 4.5. Preparation of Nanodiscs

MSP1E3D1 protein was expressed and purified [53] to generate fluorescently labeled MSP1E3D1-nanodiscs [54]. All lipids were purchased from Avanti Polar Lipids, including 18:1 1,2-dioleoyl-sn-glycero-3-phosphoethanolamine-*N*-(lissamine *rhodamine* B sulfonyl), 16:0-18:11-palmitoyl-2-oleoyl-sn-glycero-3-phosphocholine (POPC), and brain phosphatidylinositol 4,5-bisphosphate (PI(4,5)P_2_). 1.25 μmoles of lipid were used as follows: 99.95% POPC and 0.05% rhodamine phosphatidylethanolamine (PE), or 94.95% POPC, 5% PI(4,5)P_2_, and 0.05% rhodamine for [55] PI(4,5)P_2_-containing nanodiscs. The samples were dried under a continuous flow of argon. The lipids were further dried in a vacuum overnight in a desiccator. The lipids were then resuspended in 25 μL of buffer A (20 mM Tris (pH 7.5), 100 mM NaCl, 100 mM sodium cholate). The tubes were subjected to sonication until the lipid solution was clear. Then, 52.5 μL of 6 mg/mL MSP1E3D1 protein was added to obtain the final ratio of 1:130 (MSP1E3D1:lipids) and 47.5 μL of buffer A without cholate in order to obtain the final cholate concentration of 20 mM. This 125 μL nanodisc reconstitution mixture was incubated on ice for 30 min, followed by the addition of 200 mg of activated Biobeads^TM^ SM-2 resin (BioRad, Hercules, CA, USA). The tubes were gently rotated upside down overnight at room temperature to assemble the nanodiscs. The 125 μL nanodisc reconstitution mixture was then obtained from Biobeads and centrifuged at 14,000 rpm for 15 min at 4 °C. The sample was then loaded onto Superdex S-200 equilibrated with 20 mM Tris (pH 7.5), 150 mM NaCl, and 0.5 mM ethylenediaminetetraacetic acid. A total of 200 μL fractions was collected, and the peak fraction was used for further analyses.

### 4.6. Nanodisc-Plakophilin-3 Binding Assay

Nanodiscs prepared with and without 5% PI(4,5)P_2_ were incubated with plakophilin-3 (1:80 moles to moles ratio) at 4 °C for 30 min and subjected to native polyacrylamide gel electrophoresis (4-15% mini PROTEAN TGX stain-free gels, BioRad). After ultraviolet activation, the gels were visualized in the ChemiDoc Imaging system (BioRad) for 5 min.

### 4.7. Actin Polymerization

G-actin from rabbit skeletal muscle (50 μL at 2.5 mg/ml) was dialyzed against 500 mL of G-actin buffer (2 mM Tris (pH 8), 0.2 mM adenosine triphosphate, 0.2 mM CaCl_2_, 0.5 mM β-mercaptoethanol) at 4 °C. G-actin (at 1.28 mg/mL) was then polymerized by adding the polymerization buffer (50 mM KCl, 2 mM MgCl_2_, 10 mM imidazole, 2 mM adenosine triphosphate, and 5 mM ethyleneglycol-bis(β -aminoethyl)-tetra-acetic acid) for 3 h at ambient temperature. F-actin was stored at 4 °C for up to two weeks.

### 4.8. Actin Binding Assays

Proteins were centrifuged at 32,500 rpm at 4 °C for 15 min before use. For the F-actin co-sedimentation assay, the proteins were incubated with polymerized F-actin in different ratios for 30 min and centrifuged at 32,500 rpm at 4 °C for 20 min. For the F-actin bundling assay, cytoskeletal proteins were incubated with polymerized F-actin for 30 min in different ratios and centrifuged at 10,000 rpm at 4 °C for 20 min. 

### 4.9. Cryogenic Electron Microscopy Grid Preparation

Au-Flat 1.2/1.3 300 mesh grids (Protochips, Morrisville, NC, USA) were glow-discharged for 120 s at 15 mA using a PELCO easiglow (Ted Pella Inc., Redding, CA, USA) and mounted on a Leica GP2 cryogenic plunger (95% humidity and 8 °C). A total of 3.5 μL of 0.2 mg/mL (3.5 μM) plakophilin-3 was dispensed onto the carbon film side of the grid and incubated for 30 s, followed by blotting for 9 s and plunge freezing in liquid ethane, which was maintained at -183 K using liquid nitrogen.

For studies involving F-actin binding and bundling by plakophilin-3, Quantifoil grids were used. Quantifoil R 1.2/1.3 400 mesh grids were glow-discharged for 60 s at 15 mA using a PELCO easiglow (Ted Pella Inc.) and mounted on a Leica GP2 cryogenic plunger (95% humidity and 21 °C). 3.5 μL of F-actin or F-actin–protein complex (1 μM of F-actin in each case) was placed on the carbon film and incubated for 60 s, followed by blotting from the back side of the grid for a total of 15 s. The grids were immediately plunge-frozen in liquid ethane maintained at -183 K using liquid nitrogen. The grids were inserted into a JEOL cryoARM300 for screening and data collection.

### 4.10. Cryogenic Electron Microscopy Data Collection

All cryoEM data were recorded with a JEOL cryoARM300 operated at 300 kV with a K3 detector (Gatan Inc., Warrendale, PA, USA). The condenser aperture was set to 50 µm with an Omega in-column energy filter, a slit width of 20 eV, and a zero-loss peak that aligned every six hours [51]. Automated image acquisition was performed using SerialEM [56] at a nominal magnification of ×60,000 and in the super-resolution mode with a pixel size of 0.36 Å. A total electron dose of ~60 e^−^/Å^2^ at a calibrated dose of 1.2 e^−^/Å^2^ per frame was applied to the sample. Movies were recorded for 50 frames with a defocus range of −0.8 μm to −2.4 μm. Approximately 20,000 movies were collected.

### 4.11. CryoEM Data Processing

The raw movies were imported into the cryoSPARC [57] interface. Motion correction was accomplished via patch motion correction and contrast transfer function (CTF) estimation with a CTF patch estimation. The initial curation of the micrographs was carried out based on (i) a CTF fit greater than 5Å, (ii) astigmatism, and (iii) relative ice thickness.

A total of 20,105 movies were collected, and 18,220 micrographs were selected for further processing after curation. Initial blob picking was used for particle picking, followed by the particle extraction of 2,197,790 particles and the first 2D classification. The resultant two hundred 2D classes were further curated to a final number of thirty-seven 2D classes with the least noise. After two rounds of 2D classification, only 608,542 particles with good secondary features were subjected to initial ab initio volume calculation into 3 classes. All the classes were analyzed through viewing in Chimera, and the best class representing the AlphaFold model of plakophilin-3 was used for further processing. Two hundred ninety-four thousand six hundred fifty particles were subjected to multiple refinements, including heterogeneous and homogeneous refinement in cryoSPARC. The map was further improved via CTF refinement of the particles. The final homogenous refinement yielded a reconstruction with a 5 Å resolution. The directional resolution plot for the map was then generated using the remote 3DFSC processing server (3dfsc.salk.edu accessed on 20 May 2023).

### 4.12. Structure Determination

To determine the structure of the plakophilin-3, the AlphaFold model for residues 305 to 797 was used as an initial model. Using Chimera, the final map was flipped for the correct hand after visual inspection. The AlphaFold model was then docked into the cryoEM map. The structure was then subjected to flexible fitting using real-space refinement in COOT [58] and further refined through PHENIX [59] using rigid-body and real-space refinement to obtain a better map to model correlations. Due to the resolution of the final refined map, no further attempts were made to refine the coordinates.

### 4.13. Immunofluorescence Microscopy

A431 cells were cultured on glass-bottomed dishes (P35G-1.5; MatTek, Ashland, MA, USA) for 48 to 64 h as described previously [60]. The cells were fixed with 2% formaldehyde (10 min) and then permeabilized with 1% triton X-100 and stained for the corresponding antibodies [7]. Immediately before imaging, the dishes were filled with 87% glycerol. The images were taken using a Nikon A1 laser-scanning confocal microscope (the Z step size was 0. 5 mm in all cases) equipped with a Plan Apo TIRF 100×/1.45NA objective lens.

The following antibodies were used: guinea pig anti-desmoplakin (DP-1; Progen, Wayne, PA, USA); rabbit anti-β-catenin (MA5-14461, Invitrogen, Waltham, MA, USA); rabbit anti-desmoglein2 (21880-1-AP, Proteintech, Rosemont, IL, USA); and mouse anti-Pkp3 (sc-166655, Santa Cruz Biotechnology, Dallas, TX, USA). The actin cytoskeleton was visualized using Alexa Fluor 488-conjugated phalloidin (Invitrogen). The specificity of all the antibodies was tested by combining Western blotting and specific CRISPR/Cas9 knockout. All secondary antibodies were produced in Donkey (Jackson Immunoresearch Laboratories, West Grove, PA, USA).

## 5. Conclusions

Our study revealed the structure and new functions of plakophilin-3, a critical component of desmosomes. First, we found that plakophilin-3 forms homodimers potentially mediated by a loop that connects the fifth and sixth armadillo repeat motifs. Second, we also discovered that plakophilin-3 directly interacts with F-actin, suggesting a potential role in modulating the actin cytoskeleton. This finding is particularly intriguing, as it may explain the association of extra-desmosomal plakophilin-3 with the actin cytoskeleton. Third, our lipid binding analyses revealed that plakophilin-3 is recruited to the plasma membrane through interactions mediated by phosphatidylinositol 4,5-bisphosphate. This discovery suggests a new mechanism through which plakophilin-3 might participate in cell-cell adhesion. Collectively, our study provides new insights into the multifaceted properties of plakophilin-3. Our findings contribute to our understanding of the structure–function relationship of plakophilins. Follow-up investigations may aid in the development of novel therapeutic approaches targeting desmosomal proteins in cell-adhesion- and tissue-integrity-related diseases.

## Figures and Tables

**Table 1:** CryoEM data collection and processing statistics.

Data Collection and Processing	Plakophilin-3 (Residues 305-797)
magnification	60,000
total dose	60 e^−^ *per* Å^2^
frames	50
pixel size	0.72 Å *per* pixel
defocus range	−0.8 to −2.4 μm
number of micrographs	18,220
number of initial particles	608,542
number of final particles	294,650
map resolution	5.03 Å
Fourier shell correlation threshold	0.143

## Data Availability

The final 3D-reconstructed model of the plakophilin-3 monomer is deposited with the EMDB (accession code 40675). The plakophilin-3 and αE-catenin plasmids used in this study for expression and purification is deposited in Addgene (accession numbers 202599 and 203332, respectively).
